# A new cave-dwelling spider of the genus *Speleoticus* (Araneae, Nesticidae) from Sichuan, China

**DOI:** 10.3897/BDJ.11.e107751

**Published:** 2023-08-21

**Authors:** Weicheng Yang, Lianfeng He, Hao Yu, Yucheng Lin

**Affiliations:** 1 School of Life Sciences, Guizhou Normal University, Guiyang, China School of Life Sciences, Guizhou Normal University Guiyang China; 2 Key Laboratory Bio-resources and Eco-environment (Ministry of Education), College of Life Sciences, Sichuan University, Chengdu, China Key Laboratory Bio-resources and Eco-environment (Ministry of Education), College of Life Sciences, Sichuan University Chengdu China

**Keywords:** new species, morphology, diagnosis, troglobitic spider, taxonomy

## Abstract

**Background:**

*Speleoticus* Ballarin & Li, 2016 is a relatively small spider genus of the family Nesticidae, currently including five cave-dwelling species, distributed exclusively in East Asia, four of which are known from China.

**New information:**

A new troglobitic spider of the genus *Speleoticus* from the limestone Cave Hanwang Dong, north-eastern Sichuan, China, is described under the name of *S.hei* Yu & Lin, **sp. n.** Detailed descriptions, photographs and a distribution map of the new species are provided.

## Introduction

*Speleoticus* Ballarin & Li, 2016 is a small nesticid genus that is distributed exclusively in East Asia, with only five species described so far: *S.uenoi* (Yaginuma, 1972) endemic to Japan, *S.globosus* (Liu & Li, 2013), *S.libo* (Chen & Zhu, 2005), *S.navicellatus* (Liu & Li, 2013) and *S.yinchangminae* Li, 2016 from China (Liu and Li 2013, Lin et al. 2016, World Spider Catalog 2023). *Speleoticus* is a troglobitic spider genus and all known species are restricted to caves and exhibit a high level of adaptation to the troglobitic life: some species possess a distinct set of somatic characters, such as depigmentation, eyes reduction and elongation of legs (Lin et al. 2016).

The monophyly of *Speleoticus* is well defined by its genitalic characteristics (see Lin et al. (2016) for diagnoses of the genus). All *Speleoticus* species were known from both sexes and have been described in detail, alongside high quality illustrations, to allow easy species recognition (Yaginuma 1972, Chen and Zhu 2005, Yin et al. 2012, Liu and Li 2013, Lin et al. 2016).

**Figure 1.**
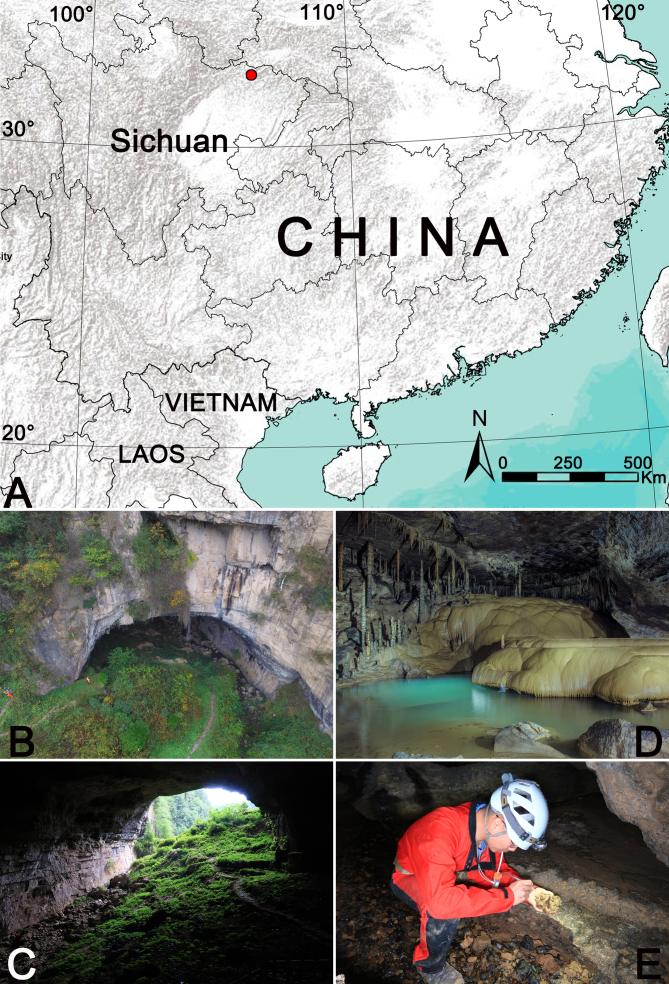
Hanwang Dong Cave, type locality of *Speleoticushei* Yu & Lin, sp. n. **A** Location of Hanwang Dong Cave; **B, C** Entrance of Hanwang Dong Cave; **D** Habitat inside the cave; **E** Collection inside the cave.

**Figure 2.**
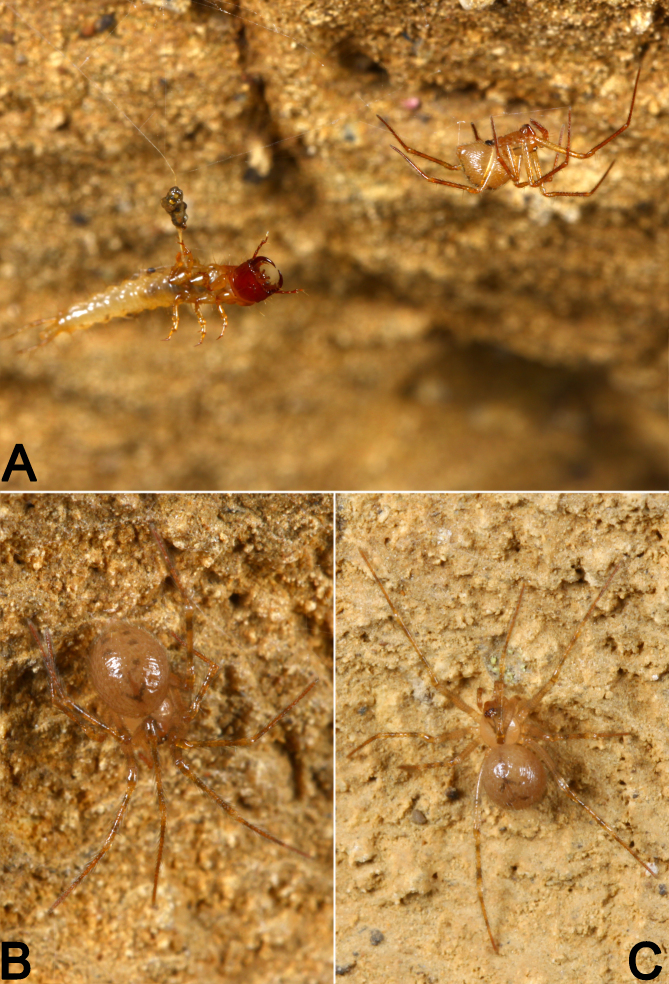
*Speleoticushei* Yu & Lin, sp. n., female paratype, live specimens. Photographs by Li He (Chengdu, Sichuan).

Recently, various expeditions to the limestone cave Hanwang Dong, north-eastern Sichuan, China (Guangyuan City: Chaotian District) (Fig. 1A–C) were carried out by colleagues of the Sichuan Cave Exploration Team (SCET, Chengdu, the cave biology group led by Mr. Li He) (Fig. 1D, E). During these surveys, we found some specimens of cave-dwelling spiders seemingly belonging to the family Nesticidae (Fig. 2). All specimens possess diagnostic characters which define them as belonging to the genus *Speleoticus*, but can be easily distinguished from the other *Speleoticus* species. The goal of this paper is to provide a detailed description and diagnosis of the new species.

## Materials and methods

Specimens in this study were hand collected. The type specimens are deposited in the Museum of Guizhou Normal University, Guiyang, Guizhou, China. Specimens were preserved in 75% alcohol and examined using an Olympus SZX7 stereomicroscope. Left male palps were examined and illustrated after dissection. Epigynes were removed and cleared in a warm 10% potassium hydroxide (KOH) solution. The vulva was photographed after being embedded in Arabic gum. Images were captured with a Canon EOS 70D digital camera (20.2 megapixels) mounted on an Olympus CX41 compound microscope and assembled using Helicon Focus 6.80 image stacking software. All measurements were obtained using an Olympus SZX7 stereomicroscope and are given in millimetres. Eye diameters were measured at the widest part. The total body length does not include the chelicerae or spinnerets. Leg lengths are given as total length (coxa, trochanter, femur, patella+tibia, metatarsus, tarsus). The terminology used in the text and figure legends follows Liu and Li (2013) and Lin et al. (2016). The distribution map was generated with ArcGIS v. 10.5 (Environmental Systems Research Institute, Inc.).

The abbreviations used in the text are: AER = anterior eye row; ALE = anterior lateral eye; AME = anterior median eye; Co = copulatory opening; Cp (I-III) = processes of the conductor (I-III) (= CDA in Liu and Li (2013)); Dp (I-II) = distal processes of the paracymbium (I-II); E = embolus; Eb = embolic base; Ma = median apophysis; MOA = median ocular area; Ms = median septum; P = paracymbium; PER = posterior eye row; PLE = posterior lateral eye; PME = posterior median eye; S = spermatheca; St = subtegulum; T = tegulum; Ta = terminal apophysis (= MA in Liu and Li (2013)); Va = ventral apophysis of the paracymbium; Vp = vulval pocket (= CD in Liu and Li (2013)).

## Taxon treatments

### Speleoticus hei

Yu & Lin sp. nov.

6B865165-A833-5B55-AE66-57BAE6F0C62F

CD180E6E-8F5C-40E0-890B-E4647E9C9FCC

#### Materials

- **Type status:** Holotype. **Occurrence:** recordedBy: Li He; individualCount: 1; sex: male; lifeStage: adult; behavior: weaver; preparations: whole animal (ETOH); occurrenceID: 4F2FECF6-4539-5B2E-88BA-A9B6C98A6BDC; **Taxon:** order: Araneae; family: Nesticidae; genus: Speleoticus; specificEpithet: *hei*; scientificNameAuthorship: Yu & Lin; **Location:** continent: Asia; country: China; countryCode: CHN; stateProvince: Sichuan; county: Guangyuan; locality: cave Hanwang Dong; decimalLatitude: 32.577297; decimalLongitude: 106.106979; **Identification:** identifiedBy: Hao Yu and Yucheng Lin; dateIdentified: 2022-03; identificationReferences: Lin et al. 2016; **Event:** samplingProtocol: by hand; samplingEffort: 10 km by foot; year: 2018; month: 4; day: 6; **Record Level:** basisOfRecord: PreservedSpecimen
- **Type status:** Paratype. **Occurrence:** recordedBy: Li He; individualCount: 5; sex: 2 males, 3 females; lifeStage: adult; behavior: weaver; preparations: whole animal (ETOH); occurrenceID: F3ACEB49-BC9E-5D14-8965-7D441B18B1AA; **Taxon:** order: Araneae; family: Nesticidae; genus: Speleoticus; specificEpithet: *hei*; scientificNameAuthorship: Yu & Lin; **Location:** continent: Asia; country: China; countryCode: CHN; stateProvince: Sichuan; county: Guangyuan; locality: cave Hanwang Dong; decimalLatitude: 32.577297; decimalLongitude: 106.106979; **Identification:** identifiedBy: Hao Yu and Yucheng Lin; dateIdentified: 2022-03; identificationReferences: Lin et al. 2016; **Event:** samplingProtocol: by hand; samplingEffort: 10 km by foot; year: 2018; month: 4; day: 6; **Record Level:** basisOfRecord: PreservedSpecimen

#### Description

**Figure 3.**
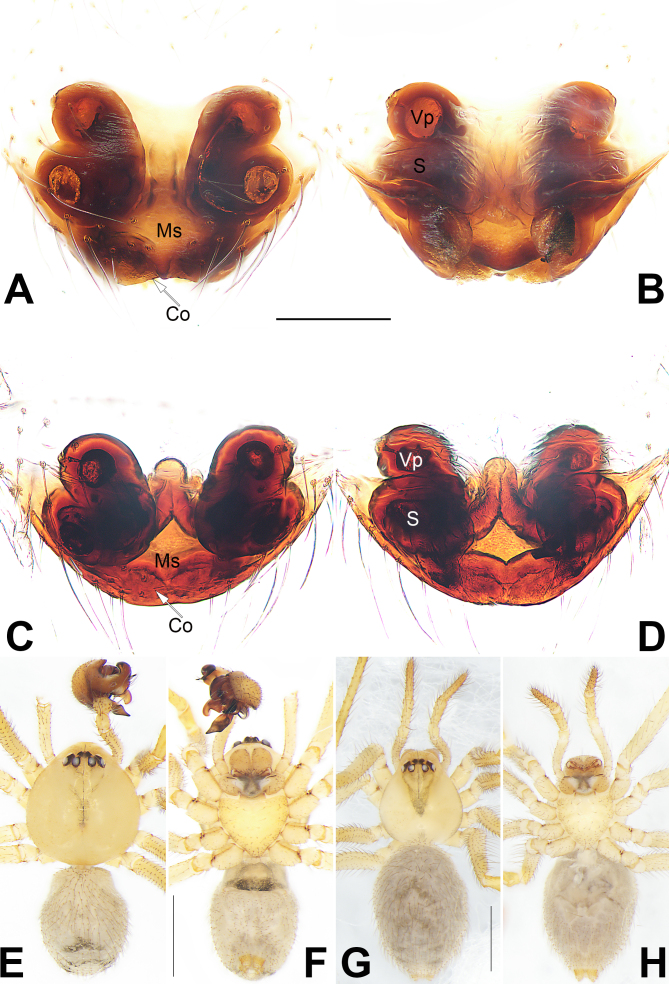
*Speleoticushei* Yu & Lin, sp. n., female paratype and male holotype. **A, B** Macerated epigyne, ventral and dorsal view; **C, D** Epigyne, macerated and embedded in Arabic gum, ventral and dorsal view; **E, F** Male, habitus, ventral and dorsal view; **G, H** Female, habitus, ventral and dorsal view. Abbreviations: Co = copulatory opening; Ms = median septum; S = spermatheca; Vp = vulval pocket. Scale bars: 0.2 mm (equal for A–D); 2 mm (equal for E–F, equal for G–H).

**Male holotype.** Habitus as in Fig. 3E and F. Total length 3.15. Carapace length 1.45, width 1.39, pale yellow to beige, with faint dark areas around the cervical furrow and fovea. Cervical groove and fovea distinct. Eyes (Fig. 3E): in dorsal view, AER slightly recurved, PER almost straight and slightly wider than AER. Eye sizes and interdistances: AME 0.09, ALE 0.09, PME 0.09, PLE 0.10; AME–AME 0.02, AME−ALE 0.05, PME–PME 0.11, PME−PLE 0.06; MOA 0.20 long, anterior width 0.19, posterior width 0.29. Mouthparts: chelicerae light orange, with three teeth on promargin; endite length 0.55, width 0.29, depressed posteriorly, slightly convergent anteriorly, with dense setae on inner margin; labium nearly rectangular, length 0.18, width 0.14. Sternum yellowish with sparse setae, length 0.75, width 0.85. Leg uniformly yellowish. Leg measurements: I 9.44 (0.43, 0.24, 2.37, 2.93, 2.33, 1.14); II 7.82 (0.48, 0.27, 2.08, 2.30, 1.80, 0.89); III 5.92 (0.43, 0.24, 1.70, 1.62, 1.26, 0.67); IV 7.96 (0.47, 0.26, 2.28, 2.38, 1.74, 0.83). Opisthosoma ovoid, 1.70 long, 1.07 wide, grey, dorsum with setae, with two indistinctly coloured chevron stripes posteriorly.

**Figure 4.**
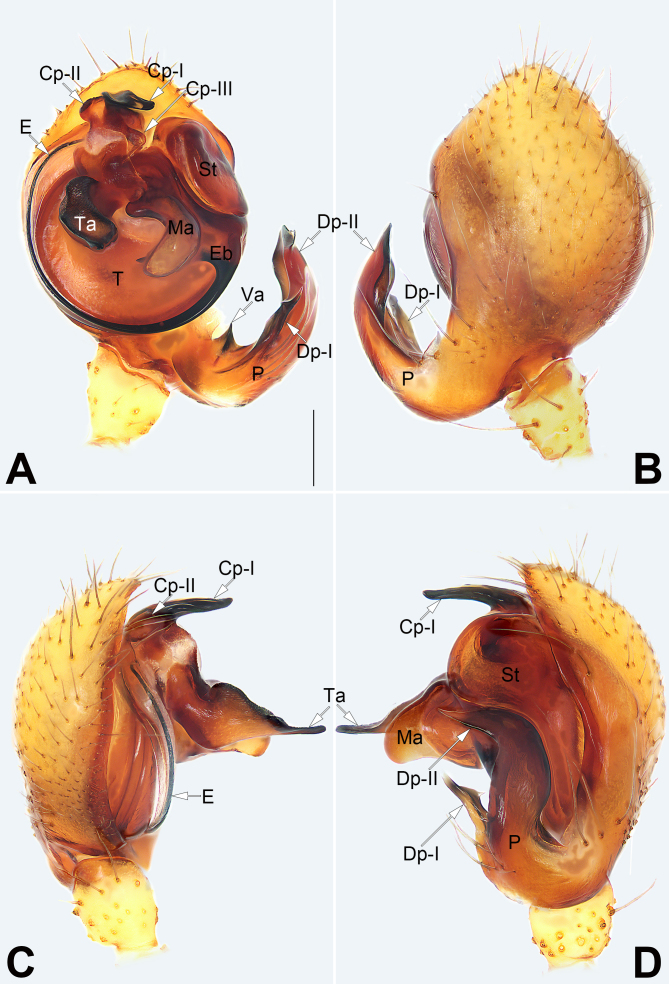
Male left palp of the holotype of *Speleoticushei* Yu & Lin, sp. n. **A** Ventral view; **B** Dorsal view; **C** Prolateral view; **D** Retrolateral view. Abbreviations: Cp (I-III) = processes of the conductor (I-III); Dp (I-II) = distal processes of the paracymbium (I-II); E = embolus; Eb = embolic base; Ma = median apophysis; P = paracymbium; St = subtegulum; T = tegulum; Ta = terminal apophysis; Va = ventral apophysis of the paracymbium. Scale bar: 0.2 mm (equal for A–D).

***Palp*** (Fig. 4A−D): Paracymbium long, with three branches or apophyses; ventral apophysis very small, shaped like a quadrahedron; Dp-I about 1/2 of Dp-II length, harpoon-shaped and with a subapical barb; Dp-II broad and long, well-developed, generally shaped like a sickle. Conductor complex, distally with three apophyses: Cp-I long and heavily-sclerotised, blade-shaped; Cp-II small, papilliform; Cp-III laminar, ending with a blunt tip. Terminal apophysis nearly triangular in prolateral view, protruding ventrally. Median apophysis heavily sclerotised, strongly expanded, directed ventrally, concave with two branches; proximal branch small, papilliform; distal branch relatively long, finger-like. Embolus slender and filiform, arising on the retrolateral flank (approximately 4 o’ clock position), surrounding the base, terminating at approximately 12 o’ clock position, its tip filiform and hidden behind the conductor.

**Female.** Colour of the living female light brown, abdomen dorsally with two ˄-shaped stripes and 3−4 pairs black spots (Fig. 2). Habitus in alcohol as in Fig. 3G and H. As in male, except as noted. Total length 3.29. length 1.31, width 1.21, pyriform. Eye diameters: AME 0.09, ALE 0.09, PME 0.09, PLE 0.07; interdistances: AME–AME 0.02, AME–ALE 0.25, PME–PME 0.12, PME–PLE 0.07; MOA 0.22 long, anterior width 0.19, posterior width 0.29. Chelicerae with three promarginal teeth. Opisthosoma: length 1.99, width 1.40, dorsum anteriorly with two ˄-shaped stripes, posteriorly with paired marks, partially fused each other in the posterior side. Leg measurements: I 8.34 (0.45, 0.25, 2.45, 2.39, 1.88, 0.93); II 6.60 (0.41, 0.23, 1.79, 1.99, 1.41, 0.77); III 5.62 (0.39, 0.22, 1.56, 1.59, 1.46, 0.41); IV 7.28 (0.45, 0.25, 2.16, 2.19, 1.53, 0.70).

***Epigyne*** (Fig. 3A−D): Epigynal plate simple, disc-shaped, scape indistinct; lateral and posterior margins indistinct, spermathecae and bursae distinctly visible through integument after dissection. Median septum short, slightly protruding, shaped as a tongue. Copulatory openings indistinct, slit like, located on the anterior margin of the cleft situated on the posterior margin of the epigynal plate. Vulva centrally with a ˄-shaped structure. Vulval pocket global, situated anteriorly, widely separated from each other by ca. 1.5 × diameters. Spermathecae situated posteriorly to vulval pocket, relatively large, oval, ca. 1.6 × longer than wide, the two spermathecae are separated by 0.6 lengths.

#### Diagnosis

Males of *S.hei* Yu & Lin, sp. n. can be distinguished from those of all other congeners by the direction of the terminal apophysis (MA in Liu and Li (2013)), which is pointed ventrally (Fig. 4A, C and D) (vs. pointed prolaterally) and by the presence of a distinct and heavily-sclerotised median apophysis (Fig. 4A and D) (vs. median apophysis absent). Amongst the species of genus *Speleoticus*, the female of the new species resembles *S.globosus* in having a similarly-shaped epigynum, but can be distinguished from the latter by: (1) copulatory openings indistinct (vs. distinct) (cf. Fig. 3A and C and Liu and Li (2013): figs. 32D and 33B); (2) vulval pocket slightly smaller than spermatheca (vs. vulval pocket distinctly larger) (cf. Fig. 3B and D and Liu and Li (2013): figs. 32B, D and 34A); (3) the presence of all eight eyes (vs. eyes absent) (cf. Fig. 3 and Liu and Li (2013): fig. 34D).

#### Etymology

This species is a patronymic named after Mr. Li He (Chengdu City, China), collector of the types, who has greatly helped us in our research.

#### Distribution

Known only from the type locality, Hanwang Dong Cave, Guangyuan, Sichuan, China (Fig. 1).

#### Biology

The types of *S.hei* Yu & Lin, sp. n. were collected under stones in the moist area about 50–500 m from the entrance in Hanwang Dong Cave.

## Supplementary Material

XML Treatment for Speleoticus hei
